# Cytochrome P450 induction properties of food and herbal-derived compounds using a novel multiplex RT-qPCR in vitro assay, a drug–food interaction prediction tool

**DOI:** 10.1002/fsn3.122

**Published:** 2014-06-10

**Authors:** Xue Fen Koe, Tengku Sifzizul Tengku Muhammad, Alexander Shu-Chien Chong, Habibah Abdul Wahab, Mei Lan Tan

**Affiliations:** 1 Malaysian Institute of Pharmaceuticals & Nutraceuticals, Ministry of Science, Technology & Innovation (MOSTI)Halaman Bukit Gambir, 11700, Georgetown, Pulau Pinang, Malaysia; 2 School of Fundamental Science, Universiti Malaysia Terengganu21030, Kuala Terengganu, Terengganu, Malaysia; 3 The Centre for Chemical Biology, Universiti Sains MalaysiaGeorgetown, Pulau Pinang, Malaysia; 4 School of Pharmaceutical Sciences, Universiti Sains MalaysiaGeorgetown, Pulau Pinang, Malaysia; 5 Advanced Medical & Dental Institute, Universiti Sains MalaysiaGeorgetown, Pulau Pinang, Malaysia

**Keywords:** CYP1A2, CYP2D6, CYP3A4, drug–food interactions, multiplex RT-qPCR

## Abstract

A multiplex RT-qPCR was developed to examine CYP1A2, CYP2D6, and CYP3A4 induction properties of compounds from food and herbal sources. The induction of drug metabolizing enzymes is an important pharmacokinetic interaction with unique features in comparison with inhibition of metabolizing enzymes. Cytochrome induction can lead to serious drug–drug or drug–food interactions, especially if the coadministered drug plasma level is critical as it can reduce therapeutic effects and cause complications. Using this optimized multiplex RT-qPCR, cytochrome induction properties of andrographolide, curcumin, lycopene, bergamottin, and resveratrol were determined. Andrographolide, curcumin, and lycopene produced no significant induction effects on CYP1A2, CYP2D6, and CYP3A4. However, bergamottin appeared to be a significant in vitro CYP1A2 inducer starting from 5 to 50 *μ*mol/L with induction ranging from 60 to 100-fold changes. On the other hand, resveratrol is a weak in vitro CYP1A2 inducer. Examining the cytochrome induction properties of food and herbal compounds help complement CYP inhibition studies and provide labeling and safety caution for such products.

## Introduction

Drug–food interactions or drug–nutrient interactions are gaining much attention recently as such interactions have the ability to influence patient outcome. These interactions need to be recognized, understood, predicted, and then managed as necessary as drug–drug interactions (Boullata 2013). Drug–food or drug–nutrient interaction is considered clinically significant if it alters therapeutic drug response and/or compromises nutrition status (Boullata and Hudson 2012; Boullata 2013). The severity of drug–food interactions can vary the same manner as drug–drug interactions. Specific food and nutrients are known to elicit changes in drug absorption, distribution, metabolism, and elimination (ADME) properties, often through specific mechanisms, and affecting the components of drug metabolism enzymes is common. For example, furanocoumarins from grapefruit juice such as bergamottin can cause irreversible inhibition of the cytochrome P450 enzyme, CYP3A4, mainly in the small intestine (Lown et al. 1997; Pirmohamed 2013). The clinical effects of grapefruit juice include a significant reduction in the presystemic metabolism of drugs metabolized by the CYP3A4 enzyme, resulting in an increase in systemic exposure, leading to adverse drug reactions and toxicity. In addition, flavonoids found in grapefruit juice such as naringin and hesperidin also inhibit the influx transporter protein family, namely organic anion transporting peptide and the overall effects include reduced bioavailability and subsequent decreased systemic and tissue concentrations of the affected drug (Dolton et al. 2012). It is said that inhibition of CYP3A4 is irreversible and it can last for longer than 3 days after ingestion of grape fruit juice until new enzyme has been synthesized in the gut wall (Pirmohamed 2013).

Cytochrome P450 (CYP) enzymes are a group of hem-containing enzymes playing a key role in the metabolism of a variety of chemically diverse compounds, including pharmaceutical agents, xenobiotics, and endogenous compounds. These enzymes are expressed in most tissues, with the highest abundance and largest number of individual CYP isoforms present in the liver. Among the CYP families, CYP1, CYP2, and CYP3 are most relevant for human drug metabolism and are responsible for the biotransformation of a large proportion of the commonly prescribed drugs in the United States (Venkatakrishnan et al. 2001; Zlokarnik et al. 2005). A large number of marketed drugs are metabolized by CYP1A2, CYP2D6, and CYP3A4 (Shimada et al. 1994; Nelson et al. 1996; Hellum et al. 2006). CYP1A2 represents about 13% CYP enzymes in the human liver and is involved in the metabolism of acetaminophen, clomipramine, and imipramine (Yan and Caldwell 2001). CYP2D6 constitutes ∼2% of total hepatic CYP and metabolizes cardiovascular drugs, antidepressants, and antipsychotics (Yan and Caldwell 2001; Zanger et al. 2004). It is an important CYP isoform as it accounts for the metabolism of more than 30% of drugs approved by the FDA, USA (Yan and Caldwell 2001; Gurley et al. 2008). CYP3A4 is the most abundant CYP isoform in adult liver and intestines, constituting about 29% of total CYP protein and catalyzes the metabolism for nearly 50% of marketed drugs, and therefore is the most important enzyme in drug metabolism (Yan and Caldwell 2001).

Drug metabolism enzymes govern drug the ADME process in the human body. Inhibition or induction of CYP enzymes by natural compounds or food components can alter the ADME process of the coadministered drugs. Inhibition of CYP enzymes will result in the increase in drug plasma level, which may lead to toxicity. In the case of grapefruit juice, it is known to increase the oral bioavailability of CYP3A4 substrates, including felodipine, verapamil, amiodarones, and a whole range of drugs (Jauregui-Garrido and Jauregui-Lobera 2012). On the other hand, enhancement of metabolic clearance by induction of CYP enzyme may lower plasma drug level to that below the therapeutic level. These interactions can lead to potentially severe and even life-threatening adverse reactions. For example, St. John's wort extract or *Hypericum perforatum* L. is among the most commonly used herbal medications in the United States and clinical reports indicate that it induces the CYP3A4 enzyme and reduces plasma concentrations of substrate drugs (Roby et al. 2000; Komoroski et al. 2004). Interactions between warfarin and St. John's wort, danshen, dong quai, ginseng, and gingko in patients on constant warfarin therapy and severe toxicity such as postoperative bleeding were reported (Zhou et al. 2007). Therefore, predicting the ability of a natural compound or food component affecting CYP expression will reduce the risk of adverse food–drug interactions and helps provide appropriate safety labeling and awareness to both consumers and healthcare professionals.

The induction of drug metabolizing enzymes is considered to be a case of pharmacokinetic interactions with unique features in comparison with inhibition of metabolizing enzymes (Hukkanen 2012). It is known that induction of a CYP enzyme is a slower process, and takes longer time to affect drug metabolism as compared to CYP inhibition, which can happen quickly. An additional concern is the increased risk of reactive metabolite toxicity due to an induction-mediated imbalance of detoxification and activation (Walsky and Boldt 2008). For example, in prodrugs, enhanced metabolic activation by induction can lead to increased toxicity, especially when the increase in metabolism of the parent compound leads to an increase in exposure of the toxic metabolite (Hukkanen 2012). In the clinical setting, the outcome of adding an inducer to the patient's established regimen can be difficult to detect (Hukkanen 2012). To determine whether a new chemical entity is a CYP inducer, the mRNA changes are to be measured (FDA 2012). According to Fahmi et al. (2010), the mRNA fold induction data results are more sensitive and informative as compared with enzymatic activity data. The RT-qPCR method is a robust and quantitative way of determining mRNA levels in assays.

In this study, a multiplex RT-qPCR assay using hydrolysis probes was developed and validated to quantitate the mRNA expression of CYP1A2, CYP2D6, and CYP3A4 in a single reaction. Subsequently, this multiplex assay was used to determine the effects of a group of food components on the cytochromes at the transcriptional level. Briefly, andrographolide is a diterpenoid component of *Andrographis paniculata* Nees, a tropical herb widely used for various health conditions. Bergamottin is a component of grapefruit juice and a known CYP3A4 inhibitor at the enzymatic level; however, its effects on the CYP1A2, 2D6, and CYP3A4 at the transcriptional level are currently unknown. Curcumin is a well-known dietary component derived from *Curcuma longa* L., a widely used spice. On the other hand, lycopene is a phytonutrient found in red fruits and vegetables, and resveratrol is a polyphenolic compound found in grape skin and peanuts. Despite public interest and widespread consumption of these food components, little is known about the ability of these components to induce cytochrome P450 expression. These compounds were evaluated systematically for their possible induction effects on CYP1A2, CYP2D6, and CYP3A4 mRNA, and protein expressions in HepG2 cells.

## Material and Methods

### Materials

Minimal essential medium (MEM), fetal bovine serum (FBS), penicillin and streptomycin solution, and 0.25% (v/v) trypsin-EDTA were purchased from Gibco (Carlsbad, CA). Sodium pyruvate, sodium bicarbonate, and nonessential amino acids were purchased from Sigma-Aldrich (St. Louis, MO). Furafylline, *β*-naphthoflavone, omeprazole, dexamethasone, ketoconazole, dimethylsulfoxide (DMSO), methanol, and natural compounds such as andrographolide, bergamottin, curcumin, lycopene, and resveratrol were purchased from Sigma-Aldrich. The iScript™ One-Step RT-PCR Kit With SYBR® Green was purchased from Bio-Rad Laboratories (Hercules, CA), QIAshredder™, RNeasy® Mini Kit, QuantiFast® Multiplex RT-PCR kit, HotStarTaq® Plus DNA polymerase, and dNTPs mix was obtained from Qiagen (Venlo, Limburg, Netherlands). Primary antibodies against CYP1A2, CYP2D6, and CYP3A4 were bought from Millipore (Billerica, MA) while the primary antibody for *β*-actin and secondary antibody (anti-rabbit IgG, HRP-linked) was obtained from Cell Signaling Technology (Beverly, MA). Primers were synthesized by Bioneer (Daejeon, Korea) and probes were synthesized by Sigma-Aldrich.

### Cell culture and treatment conditions

HepG2 cells were obtained from the American Type Culture Collection (ATCC, Manassas, VA). Cells were cultured in T25 flask in MEM (Gibco) supplemented with 10% (v/v) FBS (Gibco), 100 U/mL of penicillin, 100 *μ*g/mL of streptomycin, 1% (v/v) sodium pyruvate, 1% (v/v) sodium bicarbonate, and 1% (v/v) nonessential amino acid. Cultures were maintained in a humidified incubator supplemented with 5% (v/v) CO_2_ at 37°C. To determine the effects of control drugs and natural compounds on the mRNA expression and protein expression of CYP1A2, CYP2D6, and CYP3A4, HepG2 cells were treated with various concentrations of these compounds. Briefly, the cells were cultured in reduced-serum medium containing 0.5% (v/v) FBS for 4 h and subsequently treated with various concentrations of these compounds (0.1–25 *μ*mol/L) for 48 h. Omeprazole (50 *μ*mol/L) and dexamethasone (5 *μ*mol/L) were used as positive controls (inducers) for CYP1A2 and CYP3A4, respectively. The concentrations for the positive controls were optimized prior to the experiments. All compounds were diluted with concentrated DMSO. The concentration of DMSO in treated cells did not exceed 0.1% (v/v). Control cells were treated with DMSO (50% v/v) to yield a final concentration of 0.1% (v/v). There are no known CYP2D6 inducers; therefore, no positive controls for induction were used. However, in the western blot analysis for CYP2D6, quinidine (5 *μ*mol/L) was used as a control for CYP2D6 inhibition. After 48 h of treatment, either the total RNA or total protein was isolated and subjected to multiplex qRT-PCR analysis or western blot analysis, respectively.

### Primer and probe design

All primers and hydrolysis probe sequences were designed using Beacon Designer software, Version 7.7 (Premier Biosoft International, Palo Alto, CA) (Table 1). Nucleotide sequences for the four targets were retrieved from the National Center of Biotechnology Information (NCBI). Primer pairs for the four targets were designed to have similar melting temperature (*T*_m_). The hydrolysis probes were designed with *T*_m_ about 10°C higher than the primers. Both primers and probe sets were designed to minimize secondary structure and primer dimers (Table 1). Basic Local Alignment Search Tool (BLAST®) was performed to confirm the specificity of the primers and probes. Fluorophores for probe labeling were chosen to minimize overlaps in their emission spectra. Selected fluorophores were 6-FAM™ (6-carbonyl fluorescein), HEX™ (hexachloro-carbonyl fluorescein), Texas Red®, and Cy5®. Black Hole Quencher™ (BHQ™) was matched for each fluorophore.

**Table 1:** Primers sequences used for the RT-qPCR multiplex assay

Name	Oligonucleotide sequence (5′–3′)	Working concentration (nmol/L)	Accession number	Position	Amplification size (bp)
CYP1A2	TACTTGGAGGCCTTCATC (F)	400	NM_000761.3	1163–1180	130
	TTACGAAGACACAGCATTT(R)	400		1274–1292	
CYP2D6	ATGAGAACCTGTGCATAG (F)	400	NM_000106.4	812–829	101
	CGGATGTAGGATCATGAG (R)	400		895–912	
CYP3A4	GGTCCAGTGGGATTTATG (F)	400	NM_017460.3	429–446	82
	TTGGAGACAGCAATGATC (R)	400		493–510	
*β*-actin	ATCACCATTGGCAATGAG (F)	400	NM_001101.3	826–843	105
	GATGGAGTTGAAGGTAGTT(R)	400		912–930	

### RNA isolation and RT-qPCR assay

Cells were homogenized by centrifugation using QIAshredder™ and total RNA were isolated using the RNeasy® Mini Kit according to the manufacturer's instructions. Total RNA were quantitated using a spectrophotometer and the purity was assessed from the 260:280 nm ratio. Gel electrophoresis was carried out with 1% (w/v) agarose gel to confirm the integrity of the RNA sample. Two sharp bands of 18S and 28S rRNA indicate intact RNA. The total RNA was subjected to DNase treatment using RQ1 RNase-free DNase (Promega, Madison, WI) according to the manufacturer's protocol prior to RT-PCR.

The RT-qPCR reactions were carried out using CFX96 Real-Time PCR Detection System and analyzed with CFX Manager™ software, version 1.5 (Bio-Rad Laboratories). Melt-curve analysis was carried out using iScript™ One-Step RT-PCR Kit with SYBR® Green while the multiplex assays were carried out using QuantiFast® Multiplex RT-PCR kit according to the manufacturer's protocol. All the reactions were one-step RT-qPCR and performed using 200 ng of total RNA in a single reaction volume of 25 *μ*L.

### Optimization of multiplex RT-qPCR assay

The performances of the individual primer pairs were verified by using iScript™ One-Step RT-PCR Kit with SYBR® Green assay. Gradient RT-qPCR (duplex) was first carried out to narrow down the annealing temperature (*T*_a_) for the primer pairs, followed by melt-curve analysis to check the specificity of each primer pair. The hydrolysis probes were synthesized once the specificity of the primer pairs was confirmed. Primers and probes found to be nonspecific were redesigned and reoptimized. Subsequently, gradient RT-qPCR was carried out for the multiplex assay to select the optimum *T*_a_ to amplify the four primer pairs and probes simultaneously. Due to low expression of CYP1A2 in HepG2 cells, total RNA isolated from HepG2 cells treated with omeprazole (50 *μ*mol/L) for 48 h was used for optimization purposes. For the optimization of the multiplex assay, the amount of HotStarTaq® Plus DNA polymerase, the concentration of Mg^2+^, and dNTP mix were varied while the primer and probe concentration remained unaltered. Once the reactions were optimized, PCR amplification efficiency was determined using five points of twofold serial dilution of total RNA (500, 250, 125, 62.5, and 31.25 ng). Subsequently, all PCR assays were carried out using the corrected PCR efficiency. The thermal profile used for the amplification was 20 min at 50°C for reverse transcription, 5 min at 95°C for HotStarTaq® Plus DNA polymerase activation follow by a two-step PCR protocol for 40 cycles (15 sec of denaturation at 95°C and 30 sec at 56.1°C for combined optimized annealing-extension with fluorescent data acquisition).

### Calculation of copy number and statistical analysis

The final multiplex assay does not detect the presence of CYP1A2 mRNA expression in control (untreated) cells. Hence, to standardize the presentation of data, the mRNA expressions of the CYP isoform were represented in copy number (C_n_). A standard curve was generated from a dilution series of total RNA induced with omeprazole (50 *μ*mol/L). Since the relationship between log C_n_ and C_t_ is linear, the C_t_ values obtained were compared to the standard curve to generate the copy number for CYP1A2, CYP2D6, and CYP3A4 using interpolation. The reference gene, *β*-actin, was used to provide an internal marker of mRNA integrity within the experiment. All data were presented as mean copy number ± standard deviation of two independent experiments performed in triplicate (*n* = 6). Statistical analysis between the untreated and the treated group was calculated using one-way Anova and posthoc Dunnett's test, with an *α* value of 0.05 (GraphPad Prism software, Version 3.0, La Jolla, CA).

Relative quantification of gene expression was calculated according to the Pfaffl mathematical model, where appropriate, and the assumption of baseline C_t_ value of 33 was made for CYP1A2 in untreated control (C_t_ values undetected; C_t_ value of 33 was obtained during duplex amplification). Cells treated with DMSO served as the untreated control. However, for CYP1A2 mRNA expression, the furafylline–omeprazole-treated cells were compared to omeprazole-only-treated cells instead of untreated cells. The percentage of changes (as compared to positive controls) was calculated by dividing the fold changes for each concentration of the test compound over the fold changes of the positive control used in the experiment.

### Evaluation of multiplex qRT-PCR assay using known inducers and inhibitors and subsequent determination of the effects of food compounds on these CYP isoforms

Evaluation of the multiplex assay was carried out using several known inducers and inhibitors of the CYP isoforms. Briefly, cells were plated onto 6-wells and upon 80–90% confluence, cells were cultured in medium containing 0.5% FBS (v/v) for 4 h and subsequently treated with a series of concentrations of the following: *β*-naphthoflavone (CYP1A2 inducer), omeprazole (CYP1A2 inducer), furafylline (CYP1A2 inhibitor), dexamethasone (CYP3A4 inducer), and ketoconazole (CYP3A4 inhibitor). Similarly, resveratrol, bergamottin, andrographolide, curcumin, and lycopene were used to treat the cells as described above. The range of concentrations used was between 0.1 and 50 *μ*mol/L for all the compounds, except for ketoconazole, for which the maximum concentration used was 25 *μ*mol/L. The plates were further incubated for 48 h prior to the assessment of mRNA and protein expression as described previously. There were no known clinical inducers of CYP2D6 mRNA; therefore, it will be interesting to know if there are possible inducers for this enzyme. To determine the inhibitory effect of furafylline on CYP1A2 mRNA expression, cells were first treated with furafylline for 2 h before omeprazole treatment.

### Western blot analysis

Total protein was isolated using ProteoJET™ mammalian cell lysis buffer (Fermentas, Pittsburgh, PA) according to the manufacturer's protocol. Protein concentration was measured using Biorad D_C_ Protein Assay and Protein Standard II BSA (Bio-Rad Laboratories). The protein samples were separated using sodiumdodecyl sulphate polyacrylamide gel electrophoresis (SDS-PAGE) gel with 4% (v/v) stacking and 10% (v/v) resolving gels before transferred to Immobilon-P polyvinylidene difluoride membrane (Millipore). Membranes were first blocked with 3% (w/v) nonfat dry milk at room temperature for 2 h and probed with optimized dilution of primary antibodies against CYP1A2 (1:1000), CYP2D6 (1:2000), CYP3A4 (1:1000), and *β*-actin (1:1000), respectively, at 4°C, overnight. Secondary antibodies were probed at room temperature for 2 h. Chemiluminescence detection was performed using horseradish peroxidase-conjugated secondary antibodies and ECL™ Western Blotting Detection Reagents (GE Healthcare, Little Chalfont, Buckinghamshire, U.K.) followed by imaging using ChemiDoc™ XRS Imaging System (Bio-Rad Laboratories). Western blot analyses were carried out in duplicate for at least two independent experiments. Densitometry scanning of the bands was carried out using Image Lab software (Version 4.0; Bio-Rad). All the isoforms were normalized to *β*-actin and data were presented as relative protein expression (*n* = 4). Statistical analysis between the untreated and the treated group was calculated similarly as described earlier.

## Results

### Verification of design of primers and probes

**Figure 1:**
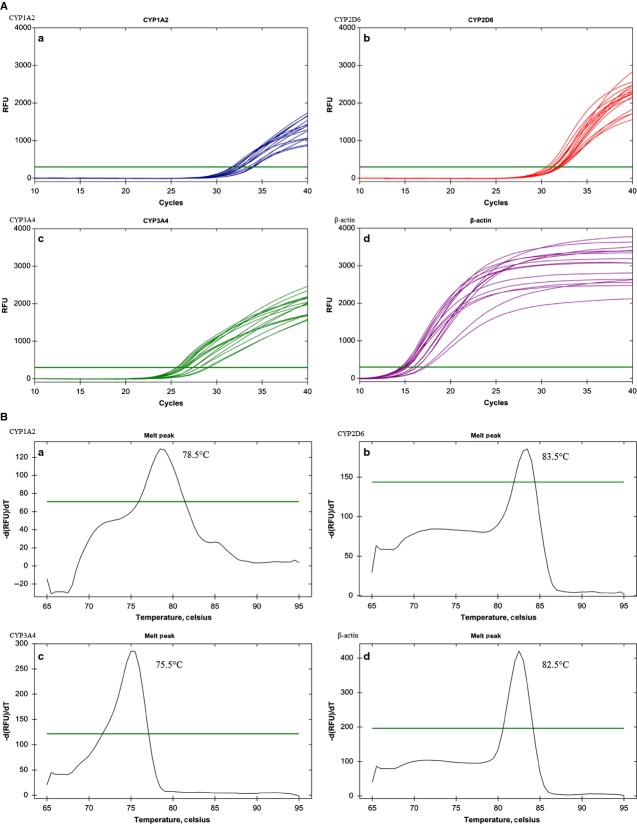
(A) PCR gradient amplification cycles for three isoforms and *β*-actin. (B) A representative melt-curve analysis of three isoforms and *β*-actin at specific temperature.

Primer and probe sequences examined using the NCBI gene BLAST algorithm (http://www.ncbi.nlm.nih.gov/blast) revealed specificity of target amplification with query coverage and maximum identity of 100%. Based on an initial gradient RT-qPCR (duplex; 46.0–60.0°C) using iScript™ One-Step RT-PCR Kit with SYBR® Green, a narrower optimum annealing temperature range (*T*_a_) was obtained (51.0–59°C). Melt-curve analysis revealed a single peak for each of the target gene indicating that the primer pairs were specific to the target and that formation of primer dimers was unlikely. *T*_m_ for all the four primer sets was near to one another (75.5–83.5°C) (Fig. 1A,B).

### Optimization of multiplex qRT-PCR assay

Subsequently, a gradient qRT-PCR (multiplex) was carried out to obtain the optimum *T*_a_ for the four targets simultaneously. The final optimum cycling condition was 20 min at 50°C for reverse transcription, followed by 5 min at 95°C for DNA polymerase activation, 15 sec at 95°C for denaturation, and 30 sec at 56.1°C for annealing, followed by extension consisting of 40 cycles/run. The final optimized multiplex assay consists of 1X QuantiFast® Multiplex RT-PCR master mix, 0.25 *μ*L/reaction of QuantiFast® RT mix, 400 nmol/L of each primer, 200 nmol/L of each probe and an additional 2 U/reaction of HotStarTaq® Plus DNA polymerase, 3 mmol/L/reaction of MgCl_2_, and 400 *μ*mol/L/reaction of dNTP. A PCR efficiency evaluation carried out using the optimized condition yielded percentage of efficiency ranging from 84.2 to 100.9°C. The PCR efficiency for CYP1A2, CYP2D6, and *β*-actin was generally more than 95% whereas for CYP3A4, it was ∼84%.

#### Effects of known inducers and inhibitors on CYP mRNA and protein expression

**Figure 2:**
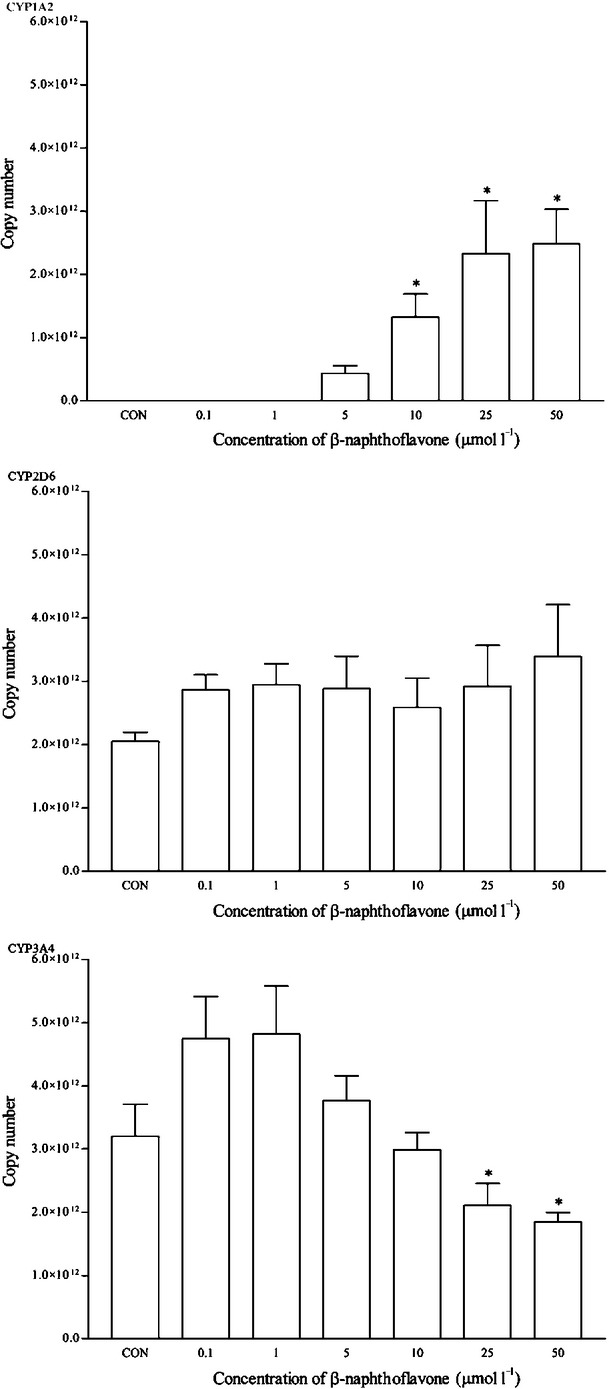
The effects of *β*-naphthoflavone on the mRNA expression of (A) CYP1A2, (B) CYP2D6, and (C) CYP3A4 in HepG2 cells after 48 h of treatment. Data are presented as mean copy number ± standard deviation of three independent experiments (*n* = 9). *P < 0.05. CON, control.

**Figure 3:**
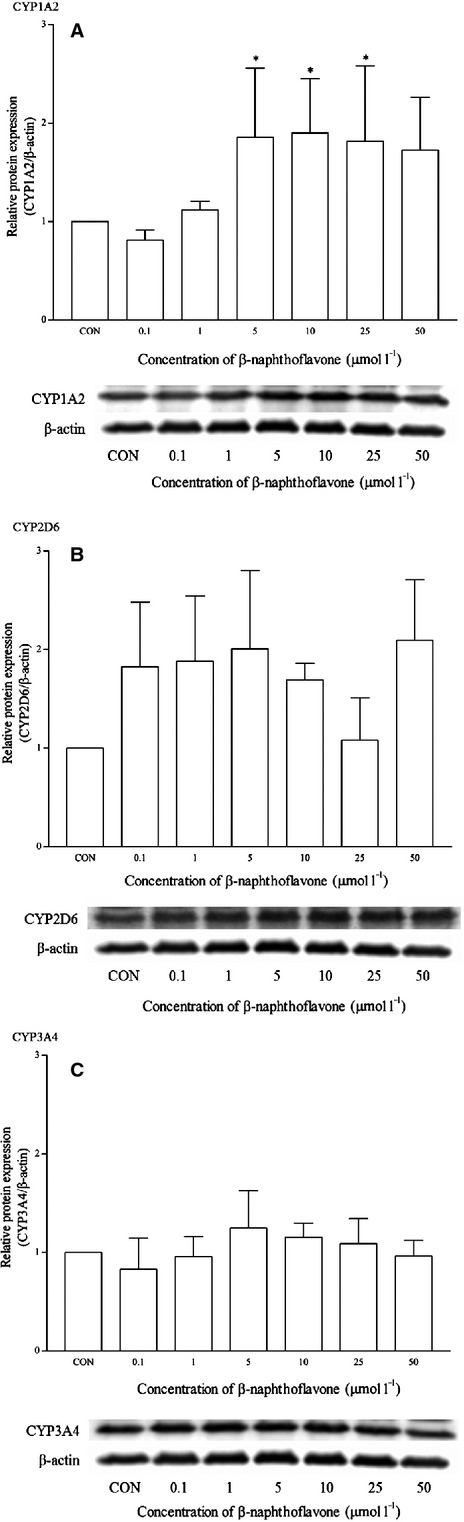
The effects of *β*-naphthoflavone on the protein expression of (A) CYP1A2, (B) CYP2D6, and (C) CYP3A4 in HepG2 cells after 48 h of treatment. Densitometry scanning data are presented as relative intensity of protein bands ± standard deviation of cytochrome over *β*-actin of two independent experiments (*n* = 4). Images of western blot analysis are representative of each isoform *P < 0.05. CON, control.

HepG2 cells were treated with various inducers or inhibitors of CYP isoforms. The mRNA expression level of CYP1A2, CYP2D6, and CYP3A4 was measured after 48 h treatment. *β*-Naphthoflavone, a known CYP1A2 inducer, up-regulates the mRNA expression of CYP1A2 as expected. A clear induction of the CYP1A2 expression was detected in the multiplex qPCR assay, where C_t_ values were obtained for *β*-naphthoflavone-treated cells starting from 5 *μ*mol/L onwards, while remaining undetected for untreated cells (Fig. 2). The mRNA expression of CYP1A2 was up-regulated significantly in a concentration-dependent manner. Based on the assumption of a C_t_ value of 33 (control; untreated), the estimated up-regulation was a more than sixfold change at 5 *μ*mol/L and went up as high as a 26-fold change at 50 *μ*mol/L. A similar pattern was observed in the protein expression of CYP1A2 (Fig. 3). The mean relative intensity was significantly higher in cells treated with 5 *μ*mol/L or higher of *β*-naphthoflavone. On the other hand, the mRNA and protein expression of CYP2D6 and CYP3A4 remained either unchanged or there were slight changes that were statistically insignificant.

**Figure 4:**
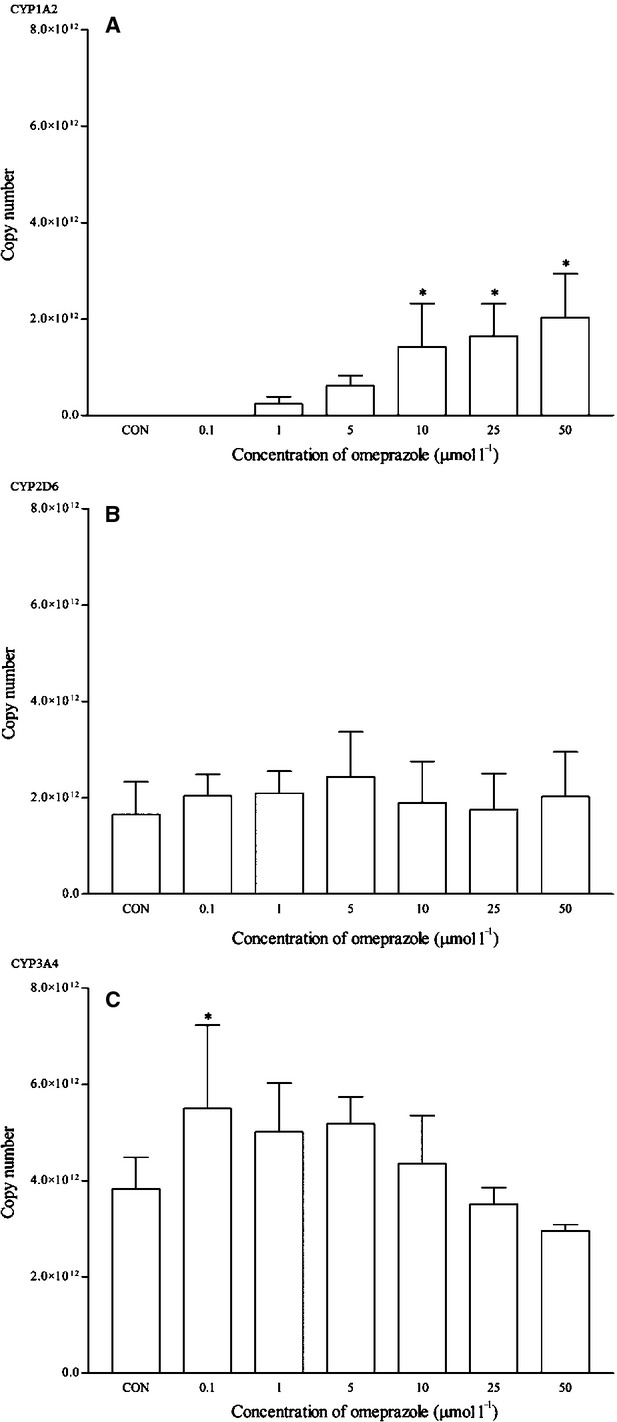
The effects of omeprazole on the mRNA expression of (A) CYP1A2, (B) CYP2D6, and (C) CYP3A4 in HepG2 cells after 48 h of treatment. Data are presented as mean copy number ± standard deviation of three independent experiments (*n* = 9) *P < 0.05. CON, control.

**Figure 5:**
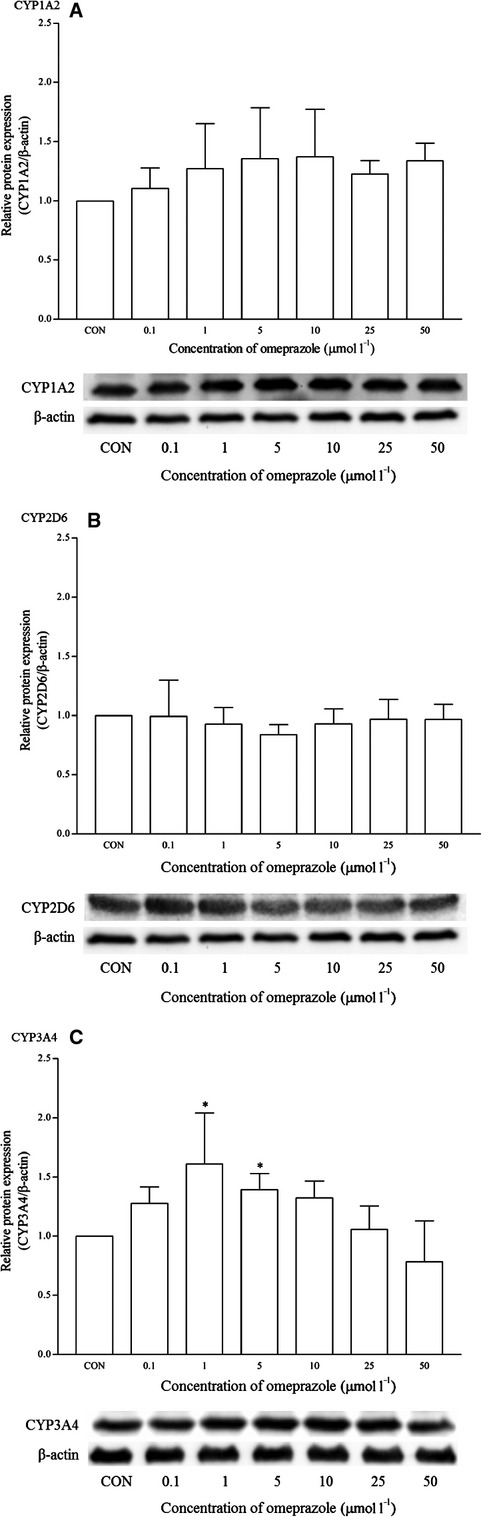
The effects of omeprazole on the protein expression of (A) CYP1A2, (B) CYP2D6, and (C) CYP3A4 in HepG2 cells after 48 h of treatment. Densitometry scanning data are presented as relative intensity of protein bands ± standard deviation of cytochrome over *β*-actin of two independent experiments (*n* = 4). Images of western blot analysis are representative of each isoform *P < 0.05. CON, control.

Omeprazole is a known clinical CYP1A2 inducer. As expected, a clear dose-dependent induction in the mRNA expression of CYP1A2 was observed and it was statistically significant from 10 *μ*mol/L onwards. CYP1A2 was up-regulated to 24-fold changes at this concentration (Fig. 4). A similar trend was observed for the CYP1A2 protein expression, even though it was statistically insignificant (Fig. 5). Omeprazole appeared to have no effects on the mRNA and protein expression of CYP2D6. However, it significantly induced the expression of CYP3A4 at 0.1 *μ*mol/L (<1.5-fold change) and protein expression at 1 and 5 *μ*mol/L, respectively.

**Figure 6:**
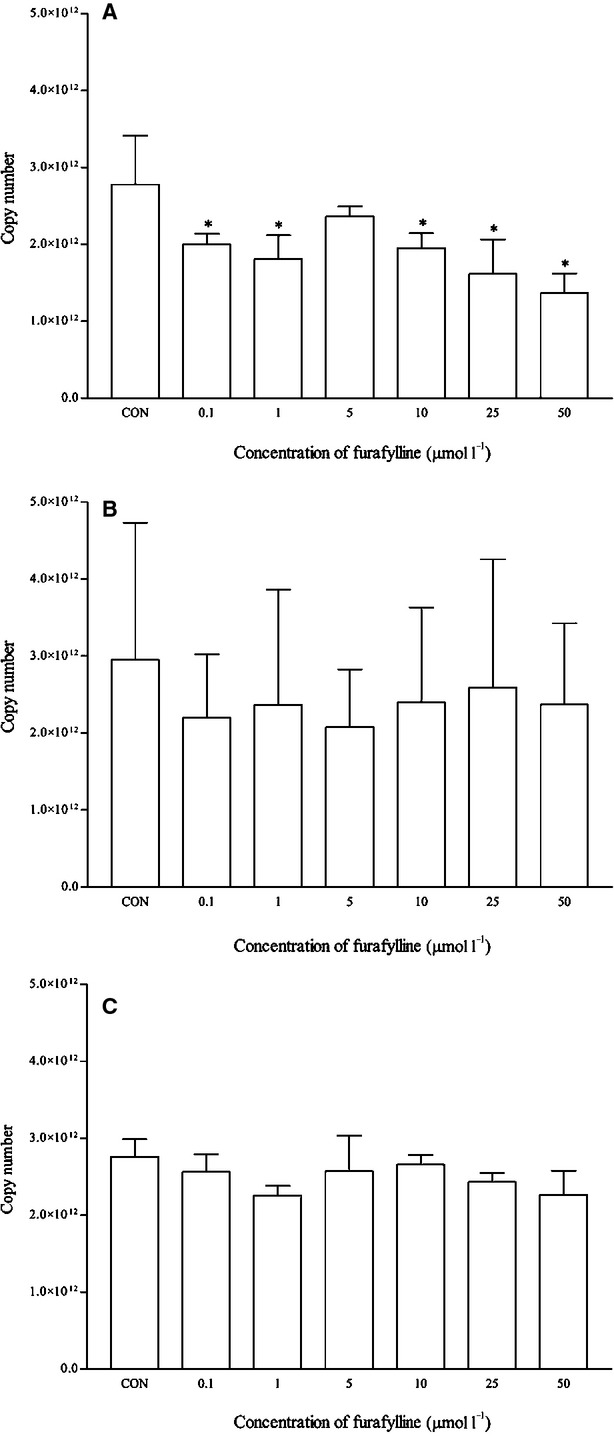
The effects of furafylline on the mRNA expression of (A) CYP1A2, (B) CYP2D6, and (C) CYP3A4 in HepG2 cells after 48 h of treatment. Data are presented as mean copy number ± standard deviation of three independent experiments (*n* = 9). *P < 0.05. CON, control; treated with omeprazole.

**Figure 7:**
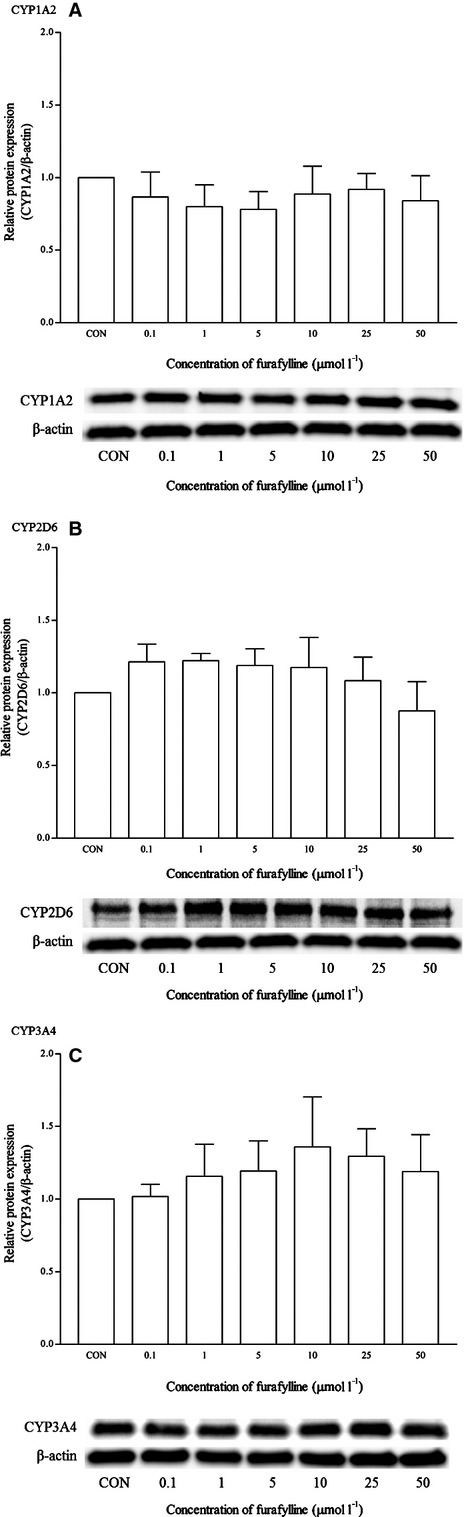
The effects of furafylline on the protein expression of (A) CYP1A2, (B) CYP2D6, and (C) CYP3A4 in HepG2 cells after 48 h of treatment. Densitometry scanning data are presented as relative intensity of protein bands ± standard deviation of cytochrome over *β*-actin of two independent experiments (*n* = 4). Images of western blot analysis are representative of each isoform. CON, control; treated with omeprazole.

Furafylline is a known inhibitor for CYP1A2 enzymatic activity. However, it is not known if furafylline inhibits the mRNA expression of CYP1A2. Interestingly, when the cells were concomitantly treated with furafylline and omeprazole, furafylline attenuated the ability of omeprazole to induce CYP1A2. The mRNA expression of CYP1A2 was significantly reduced in cells concomitantly treated with furafylline (at most concentrations) and omeprazole as compared with omeprazole-treated cells (control) (Fig. 6). However, the same cannot be said for the corresponding protein expression as the reduction was statistically insignificant (Fig. 7). On the other hand, no significant changes were observed for the rest of the isoforms.

**Figure 8:**
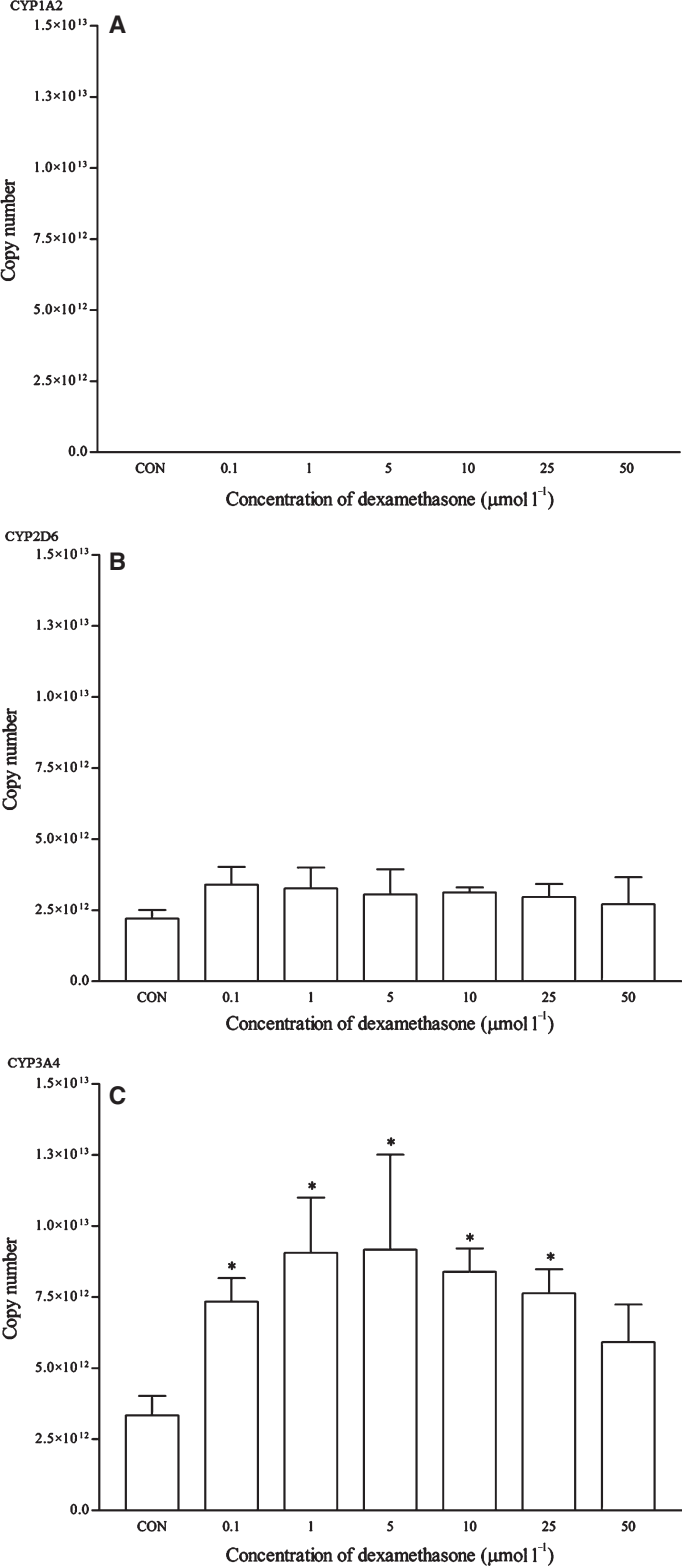
The effects of dexamethasone on the mRNA expression of (A) CYP1A2, (B) CYP2D6, and (C) CYP3A4 in HepG2 cells after 48 h of treatment. Data are presented as mean copy number ± standard deviation of three independent experiments (*n* = 9). *P < 0.05. CON, control.

**Figure 9:**
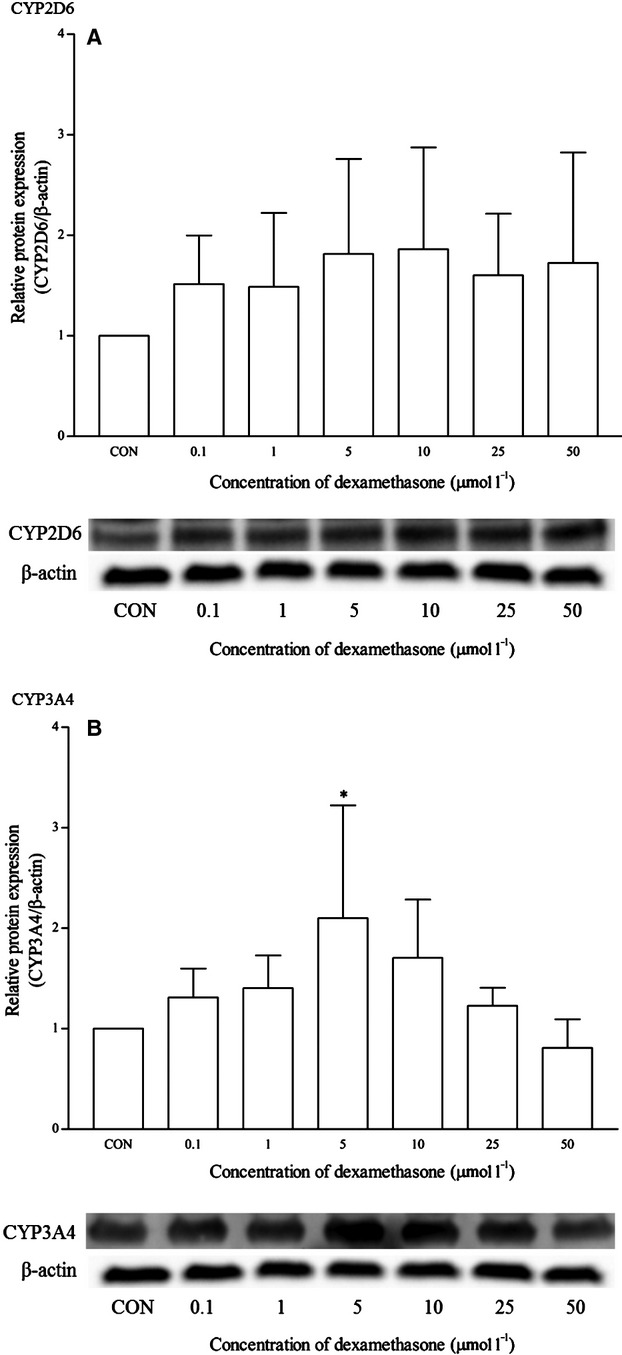
The effects of dexamethasone on the protein expression of (A) CYP2D6 and (B) CYP3A4 in HepG2 cells after 48 h of treatment. Densitometry scanning data are presented as relative intensity of protein bands ± standard deviation of cytochrome over *β*-actin of two independent experiments (*n* = 4). Images of western blot analysis are representative of each isoform *P < 0.05. CON, control.

**Figure 10:**
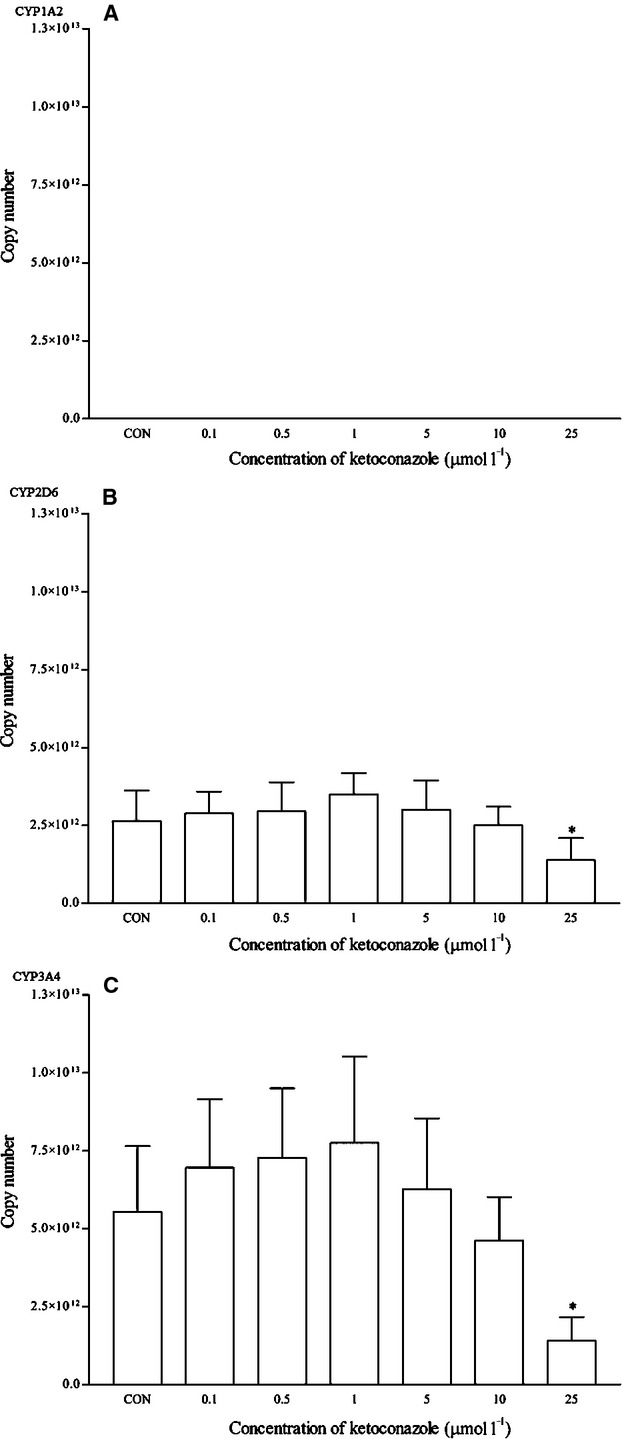
The effects of ketoconazole on the mRNA expression of (A) CYP1A2, (B) CYP2D6, and (C) CYP3A4 in HepG2 cells after 48 h of treatment. Data are presented as mean copy number ± standard deviation of three independent experiments (*n* = 9). *P < 0.05. CON, control.

**Figure 11:**
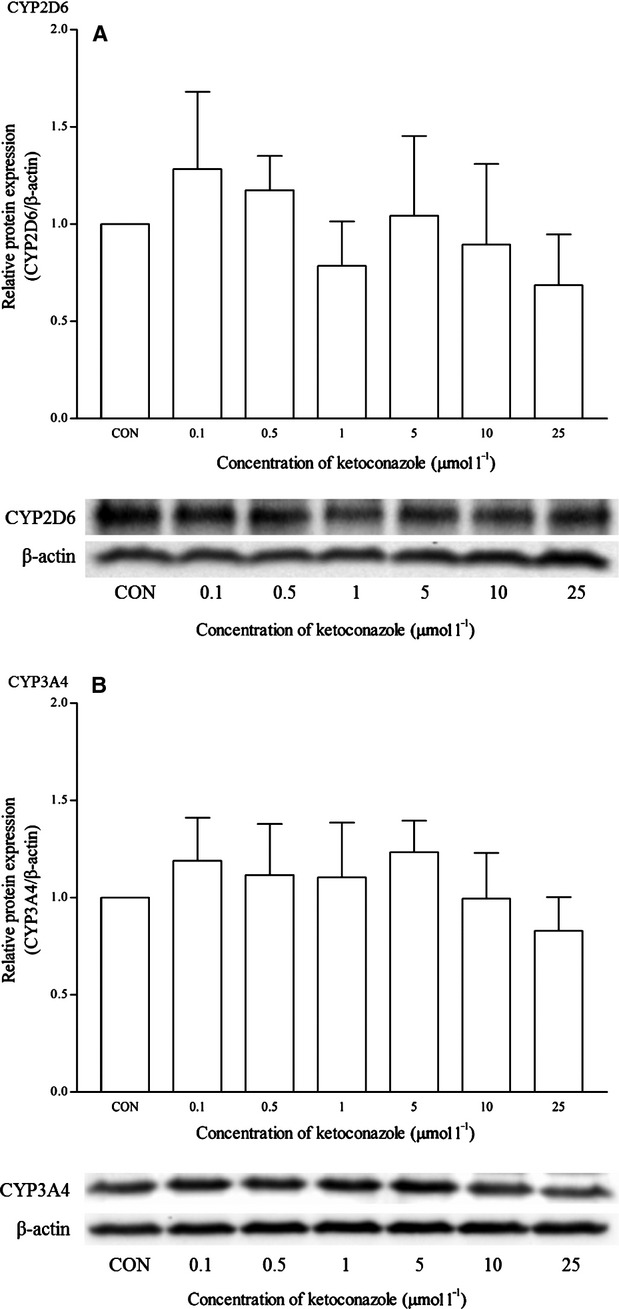
The effects of ketoconazole on the protein expression of (A) CYP2D6 and (B) CYP3A4 in HepG2 cells after 48 h of treatment. Densitometry scanning data are presented as relative intensity of protein bands ± standard deviation of cytochrome over *β*-actin of two independent experiments (*n* = 4). Images of western blot analysis are representative of each isoform. CON, control.

Dexamethasone is a known clinical inducer of CYP3A4. As expected, dexamethasone significantly induced the mRNA expression of CYP3A4 from 0.1 *μ*mol/L and onwards. Dexamethasone at 5 *μ*mol/L produced the highest induction at approximately twofold changes (Fig. 8). This was also observed in the CYP3A4 protein expression at the same concentration (Fig. 9). The expression of other isoforms was not affected by this drug. Ketoconazole is the known clinical inhibitor of the CYP3A4 enzyme and its activities on mRNA expression are largely uncertain. As seen in Figure 10, the effects of ketoconazole on the mRNA expression of CYP3A4 are largely insignificant except at the highest concentration, that is 25 *μ*mol/L. Although there was a similar trend for the corresponding protein expression, the differences were not proved to be significant (Fig. 11). A significant reduction in CYP2D6 mRNA expression was also noticed at the same concentration; otherwise, it remained insignificant.

#### Effects of food and herbal compounds on CYP mRNA and protein expression

**Figure 12:**
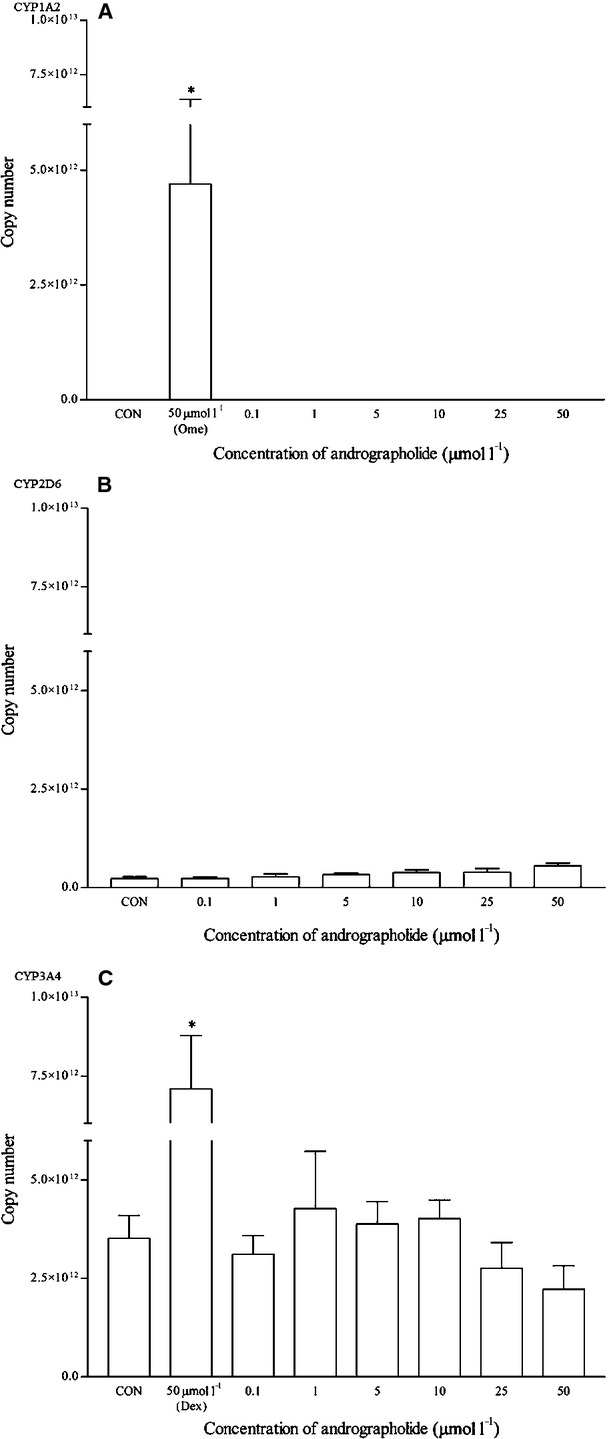
The effects of andrographolide on the mRNA expression of (A) CYP1A2, (B) CYP2D6, and (C) CYP3A4 in HepG2 cells after 48 h of treatment. Data are presented as mean copy number ± standard deviation of three independent experiments (*n* = 9). *P < 0.05. CON, control; Ome, omeprazole; Dex, dexamethasone.

**Figure 13:**
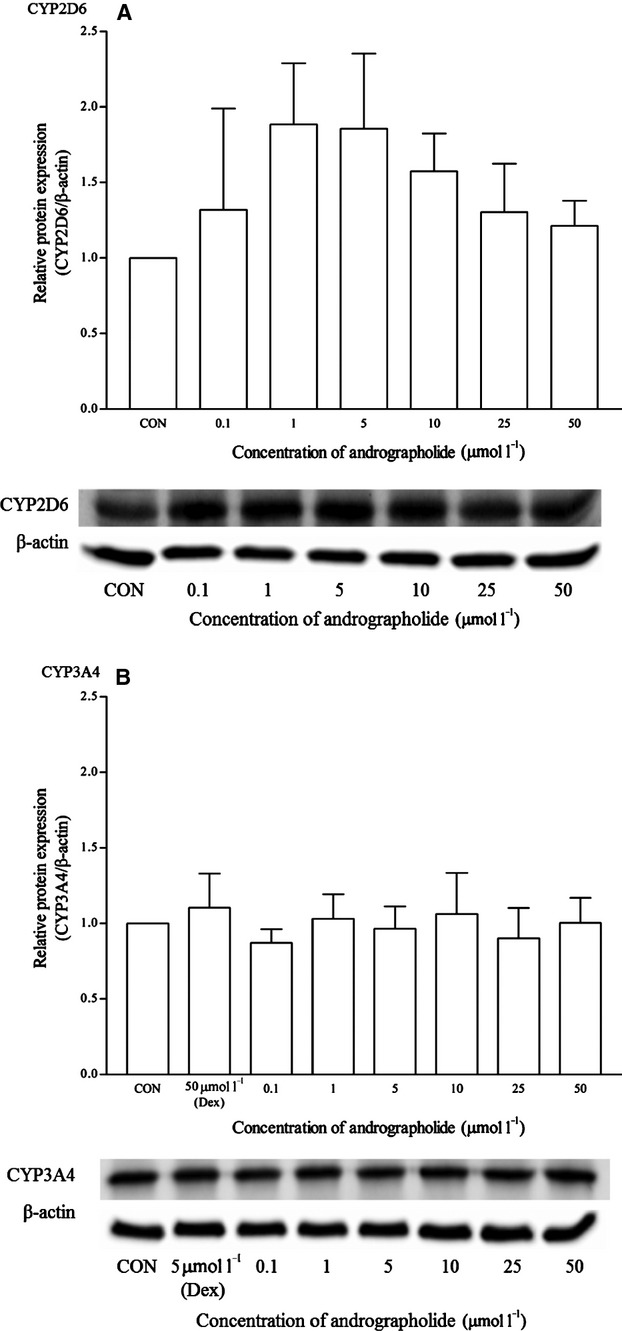
The effects of andrographolide on the protein expression of (A) CYP2D6, and (B) CYP3A4 in HepG2 cells after 48 h of treatment. Densitometry scanning data are presented as relative intensity of protein bands ± standard deviation of cytochrome over *β*-actin of two independent experiments (*n* = 4). Images of western blot analysis are representative of each isoform. CON, control; Dex, dexamethasone.

Andrographolide at all concentrations produced no effects on the mRNA expression of CYP1A2, as no amplifications or C_t_ values were detected (Fig. 12). Interestingly, CYP2D6 protein expression was up-regulated within the range of concentration tested with significant values from 1 to 5 *μ*mol/L, even though the up-regulation of the mRNA levels was not statistically significant (Figs. 12, 13). The mean up-regulation of the CYP2D6 protein was all less than twofold changes. As for CYP3A4, andrographolide did not affect both mRNA and protein expression.

**Figure 14:**
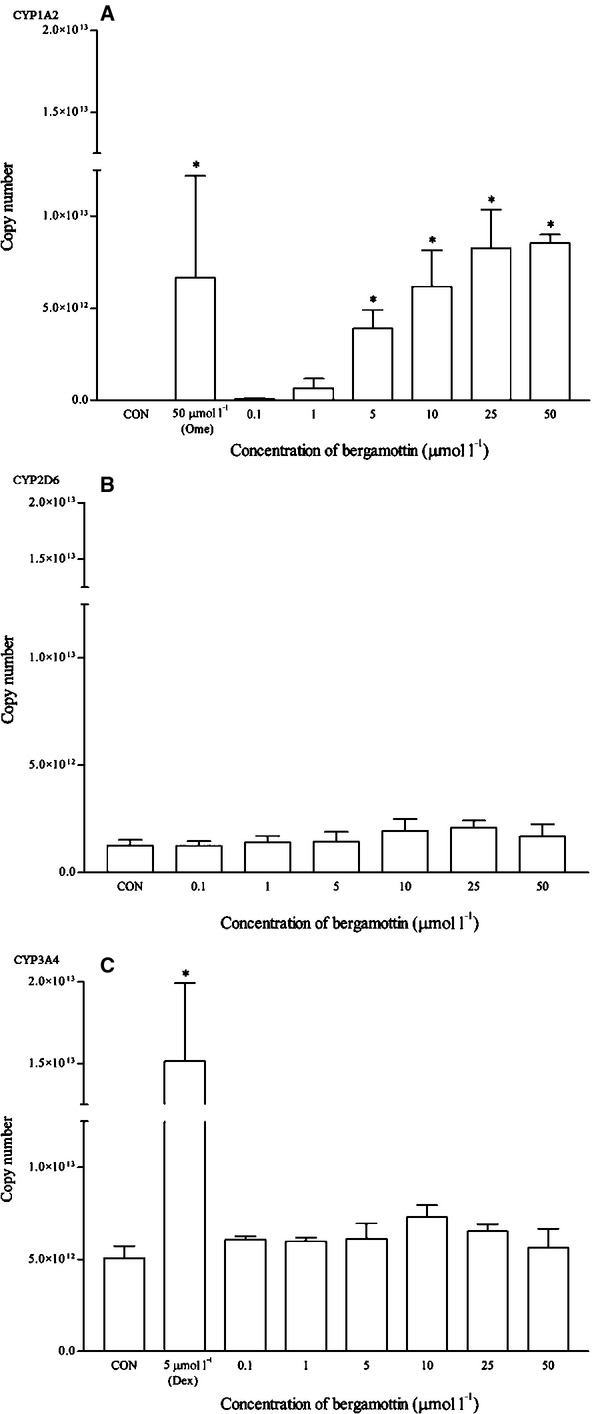
The effects of bergamottin on the mRNA expression of (A) CYP1A2, (B) CYP2D6, and (C) CYP3A4 in HepG2 cells after 48 h of treatment. Data are presented as mean copy number ± standard deviation of three independent experiments (*n* = 9). *P < 0.05. CON, control; Ome, omeprazole; Dex, dexamethasone.

**Figure 15:**
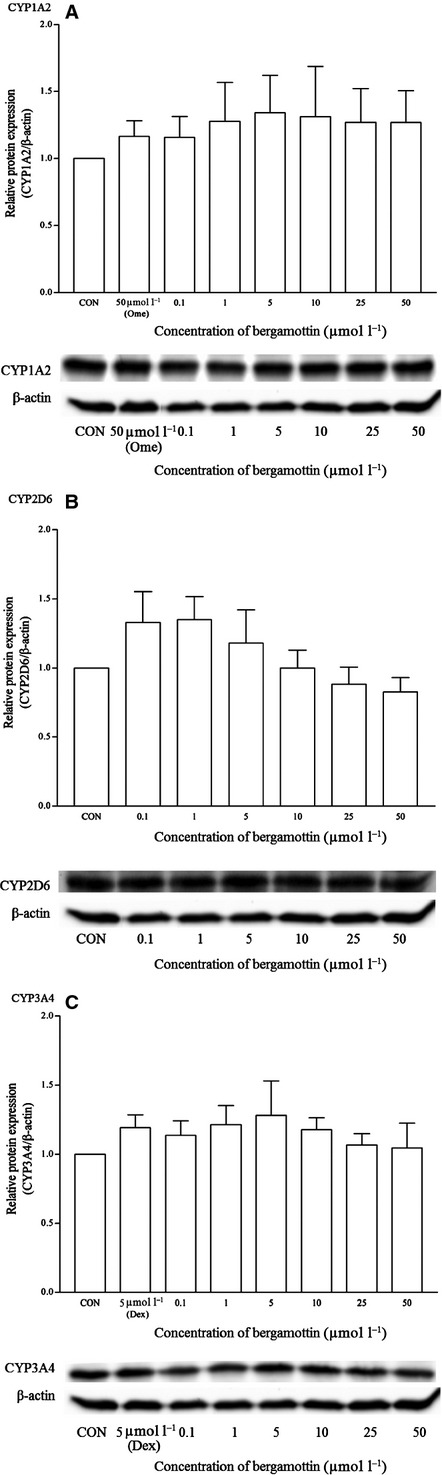
The effects of bergamottin on the protein expression of (A) CYP1A2, (B) CYP2D6, and (C) CYP3A4 in HepG2 cells after 48 h of treatment. Densitometry scanning data are presented as relative intensity of protein bands ± standard deviation of cytochrome over *β*-actin of two independent experiments (*n* = 4). Images of western blot analysis are representative of each isoform. CON, control; Ome, omeprazole; Dex, dexamethasone.

On the other hand, bergamottin significantly induced the mRNA of CYP1A2 from 5 to 50 *μ*mol/L (ranging from 60 to 100-fold changes) (Fig. 14). Interestingly, only a slight induction of CYP1A2 protein was observed and the magnitudes were statistically insignificant (Fig. 15). Bergamottin did not affect the expression of CYP2D6 and CYP3A4. Dexamethasone (5 *μ*mol/L) too did not significantly induce the protein expression of CYP3A4 as compared with the RT-qPCR and earlier western blot results.

**Figure 16:**
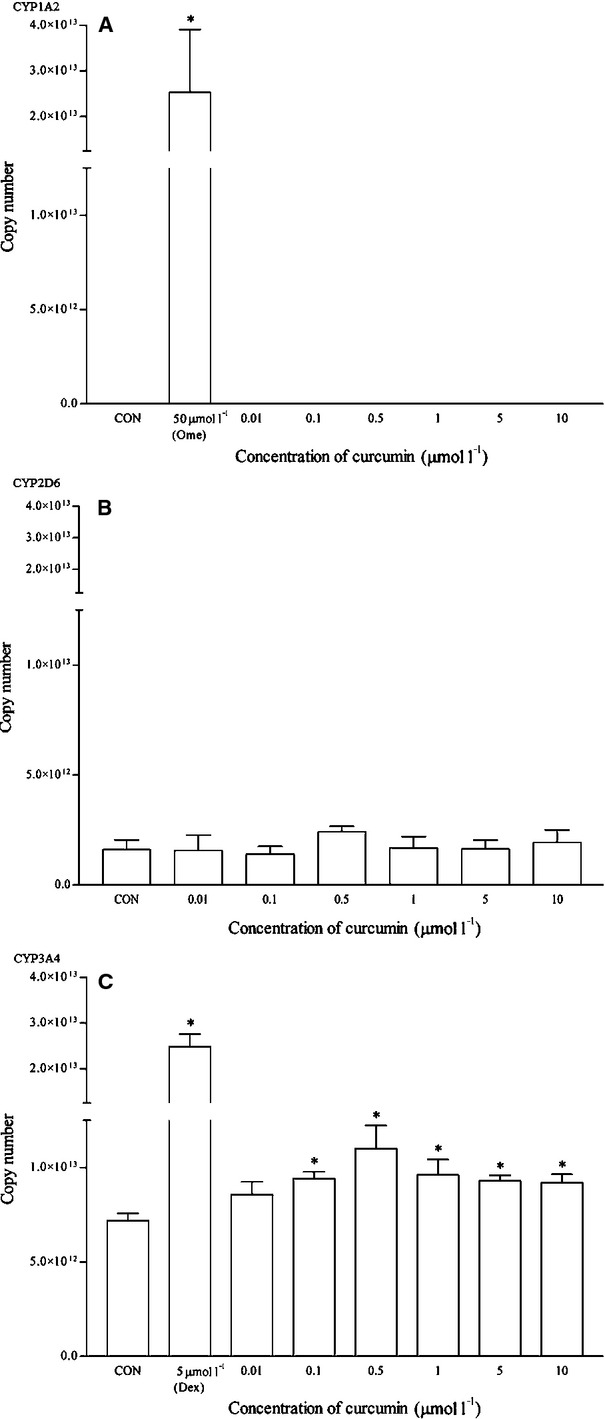
The effects of curcumin on the mRNA expression of (A) CYP1A2, (B) CYP2D6, and (C) CYP3A4 in HepG2 cells after 48 h of treatment. Data are presented as mean copy number ± standard deviation of three independent experiments (*n* = 9). *P < 0.05. CON, control; Ome, omeprazole; Dex, dexamethasone.

**Figure 17:**
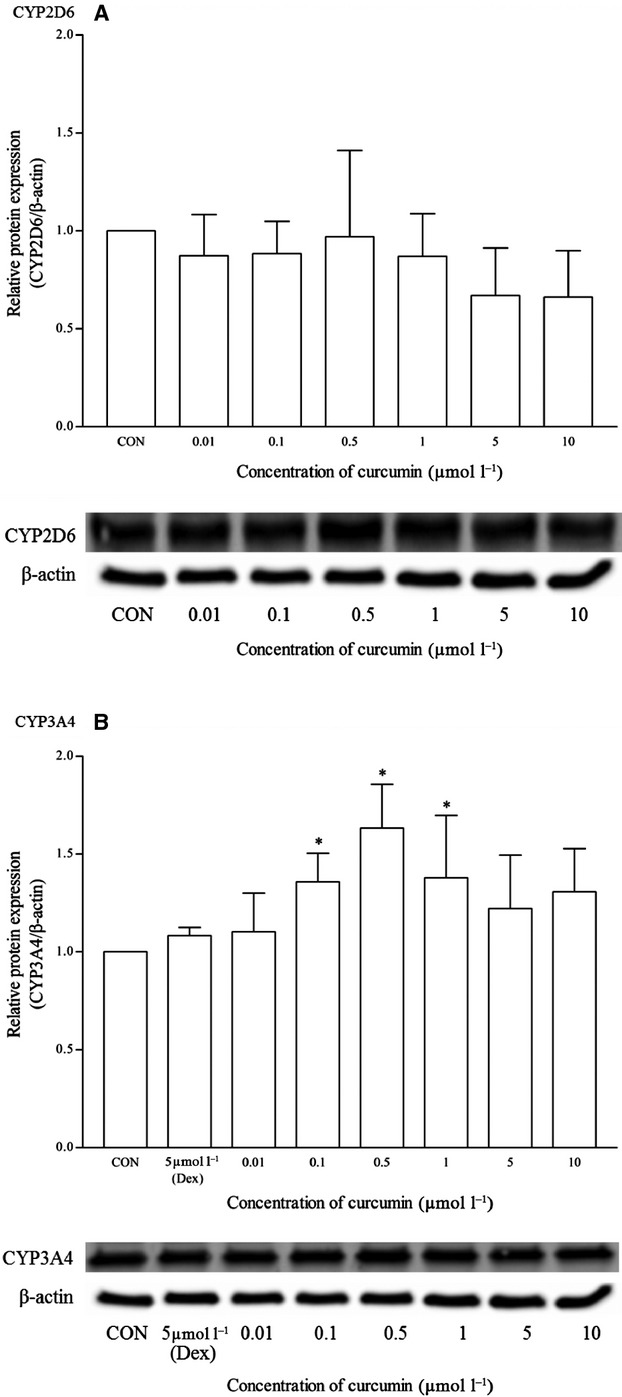
The effects of curcumin on the protein expression of (A) CYP2D6, and (B) CYP3A4 in HepG2 cells after 48 h of treatment. Densitometry scanning data are presented as relative intensity of protein bands ± standard deviation of cytochrome over *β*-actin of two independent experiments (n = 4). Images of western blot analysis are representative of each isoform *P < 0.05. CON, control; Dex, dexamethasone.

Similar to andrographolide, curcumin-treated cells did not exhibit detectable levels of CYP1A2 mRNA (Fig. 16). It does not affect significantly the mRNA and protein expression of CYP2D6 as well. However, there was significant induction of both mRNA and protein expression of CYP3A4 between the concentration range of 0.1 and 1 *μ*mol/L (Figs. 16, 17). However, the mRNA induction at all concentrations was <20% as compared to dexamethasone. On the other hand, the relative protein expression of CYP3A4 was also less than twofold changes for the same range of concentrations.

**Figure 18:**
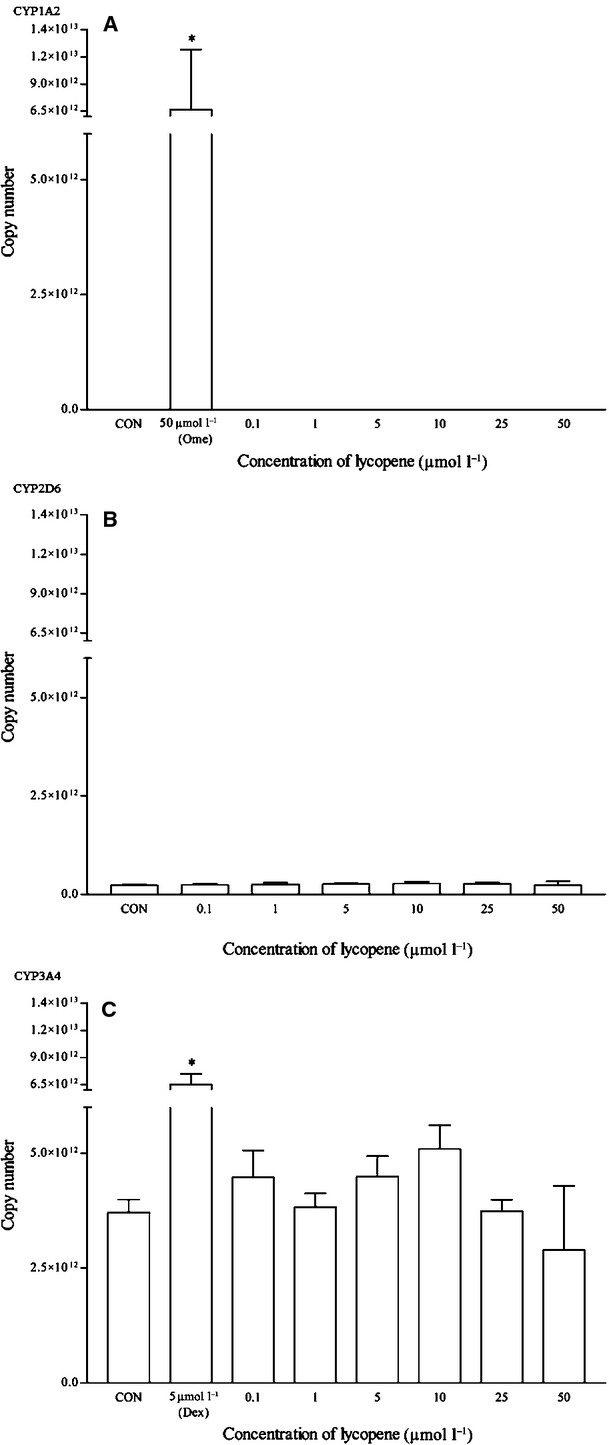
The effects of lycopene on the mRNA expression of (A) CYP1A2, (B) CYP2D6 and (C) CYP3A4 in HepG2 cells after 48 h of treatment. Data are presented as mean copy number ± standard deviation of three independent experiments (*n* = 9). *P < 0.05. CON, control; Ome, omeprazole; Dex, dexamethasone.

**Figure 19:**
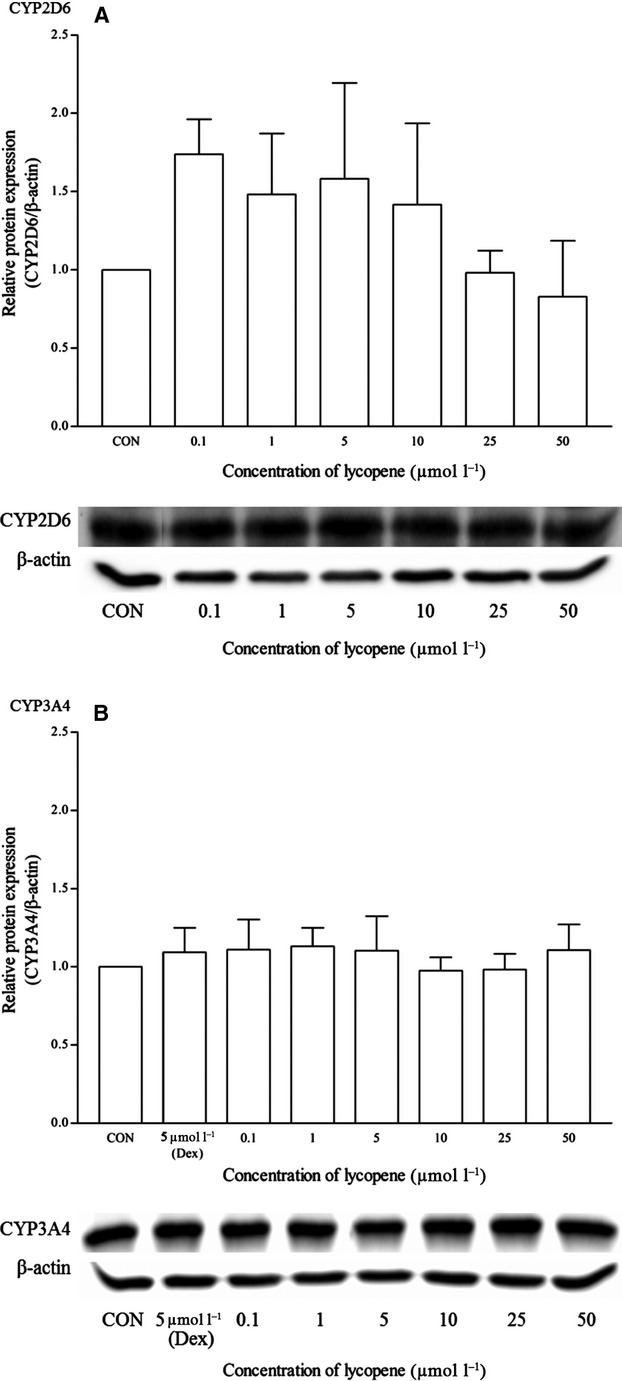
The effects of lycopene on the protein expression of (A) CYP2D6 and (B) CYP3A4 in HepG2 cells after 48 h of treatment. Densitometry scanning data are presented as relative intensity of protein bands ± standard deviation of cytochrome over *β*-actin of two independent experiments (*n* = 4). Images of western blot analysis are representative of each isoform. CON, control; Dex, dexamethasone.

**Figure 20:**
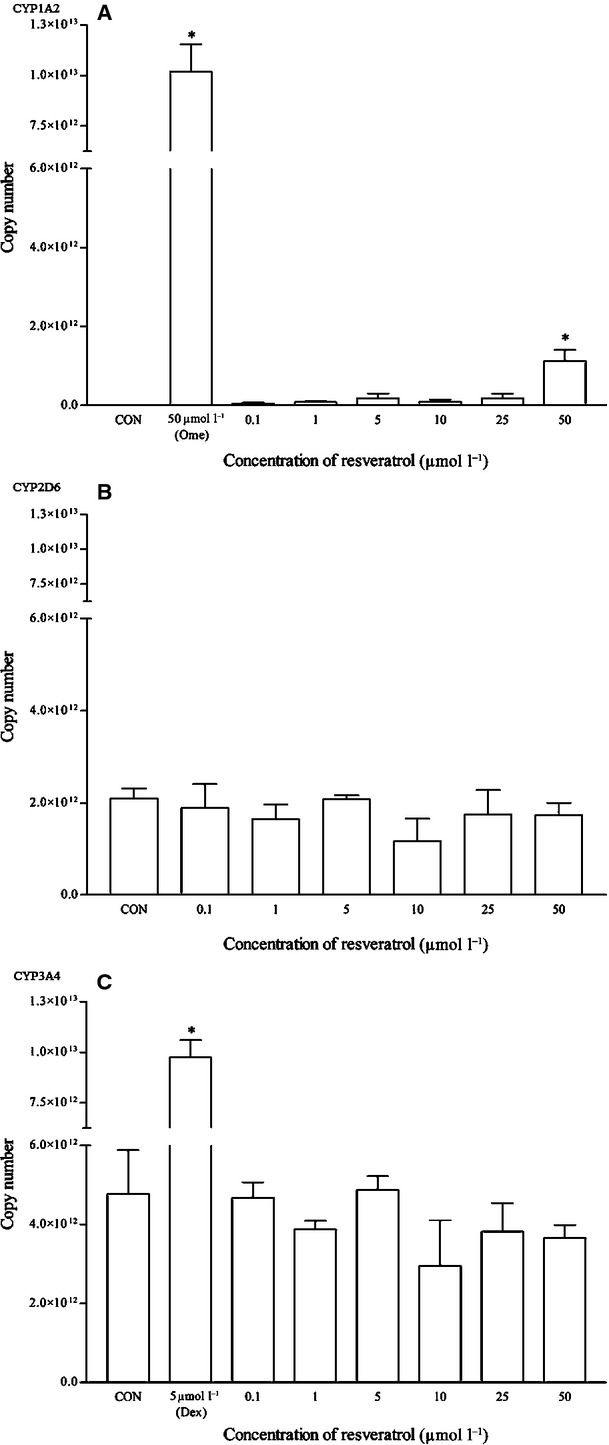
The effects of resveratrol on the mRNA expression of (A) CYP1A2, (B) CYP2D6, and (C) CYP3A4 in HepG2 cells after 48 h of treatment. Data are presented as mean copy number ± standard deviation of three independent experiments (*n* = 9). *P < 0.05. CON, control; Ome,omeprazole; Dex, dexamethasone.

Lycopene did not appear to have any effects on the mRNA and protein expression of all the isoforms (Figs. 18, 19). As for resveratrol, only the highest concentration of 50 *μ*mol/L elicited a significant mRNA CYP1A2 induction but remains insignificant at the protein expression level (Fig. 20). Resveratrol has no significant effects on both CYP2D6 and CYP3A4 mRNA expression, even though the CYP2D6 protein expression pattern suggests otherwise (Figs. 20 21). A statistically significant CYP2D6 protein induction was observed from 0.1 to 5 *μ*mol/L in resveratrol-treated cells, with more than twofold changes in average at 0.1 *μ*mol/L. Similarly, with the rest of the experiments, dexamethasone does not appear to produce a significant protein expression of CYP3A4 using western blot analysis.

**Figure 21:**
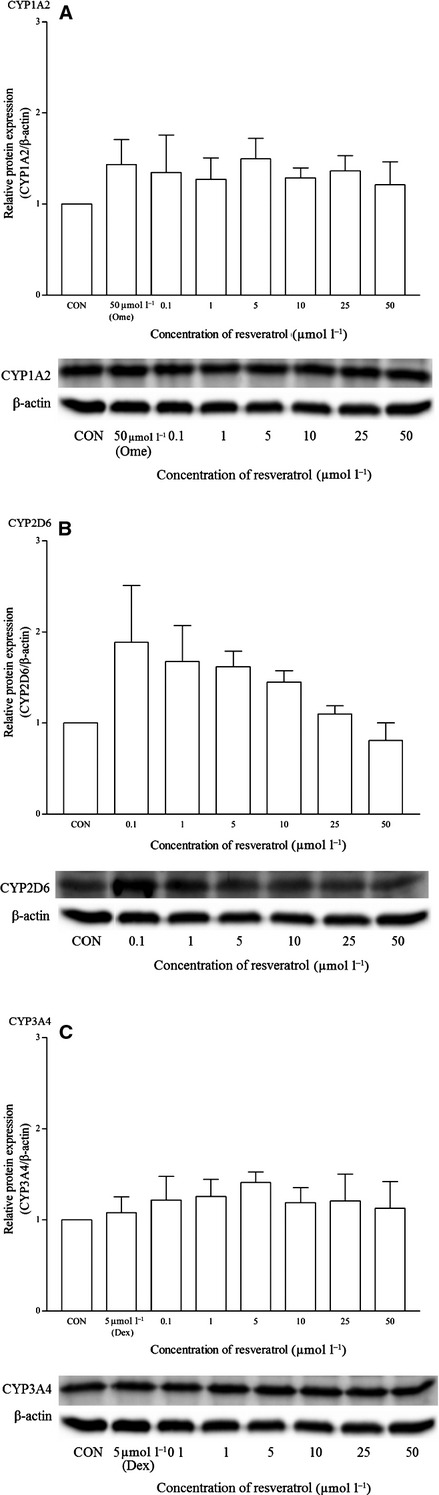
The effects of resveratrol on the protein expression of (A) CYP1A2, (B) CYP2D6, and (C) CYP3A4 in HepG2 cells after 48 h of treatment. Densitometry scanning data are presented as relative intensity of protein bands ± standard deviation of cytochrome over *β*-actin of two independent experiments (*n* = 4). Images of western blot analysis are representative of each isoform. CON, control; Ome, omeprazole; Dex, dexamethasone.

## Discussion

Quantitative real-time PCR utilizing hydrolysis probes is an indirect assay system which yields fluorescence signals during primer extension in PCR amplification. It is a robust and quantitative way of determining mRNA levels in assays. In this study, a multiplex qRT-PCR was developed to simultaneously detect three major CYP isoforms and a reference gene. This assay was able to quantify the effects of various chemical entities on the mRNA expression of CYP isoforms in a rapid and economical manner. Cytochrome P450 enzymes play important roles in the metabolism of a variety of chemically diverse compounds and xenobiotics (Bowen et al. 2000; Wrighton et al. 2000). Pertubations in CYP metabolism can lead to drug interactions which may be clinically fatal, leading to significant toxicity or resulting in treatment failures (Rodeiro et al. 2008). Drug interaction mechanisms involving the CYP enzymes are categorized as either CYP enzyme inhibition or CYP enzyme induction. Although many important drug interactions are resulted from CYP450 inhibition, the effect on CYP450 induction-mediated interaction is another major concern for the healthcare industry (Lin 2006). Unlike inhibition of CYP450, induction is a slow regulatory process and occurs at the transcriptional level. CYP450 induction causes rapid CYP450 metabolism activity of a coadministered drug, resulting in subtherapeutic drug concentration and a reduction in therapeutic efficacy, which can lead to life-threatening incidences (Lin 2006). Xenobiotics in general have the ability to induce the mRNA expression of CYP isoforms and according to the FDA draft guideline for drug–drug interaction studies, the cytochrome induction properties of any xenobiotics or compounds can be determined by examining their mRNA levels in suitable test models (FDA 2006, 2012). Fahmi et al. (2010) stated that a fourfold and twofold cutoff of a mean fold maximum increase in CYP450 mRNA and enzymatic activity from human hepatocytes produced the lowest rates of false negatives as compared with using a cutoff maximum induction achieving 20–40% of the effects of a positive control. These criteria could be used as cutoff points for in vitro studies before proceeding to clinical trials.

In order to evaluate the multiplex qRT-PCR assay, mRNA expression profiles of CYP1A2, CYP2D6, and CYP3A4 by both known inducers and inhibitors were determined. Omeprazole and *β*-naphthoflavone are known inducers for CYP1A2 which induce the transcription of CYP1A2 via AhR activation (Diaz et al. 1990; Madan et al. 2003; Westerink and Schoonen 2007). As expected, both positive controls induced CYP1A2 mRNA expression and these were clearly observed from the multiplex RT-qPCR profile. Although omeprazole induced the mRNA expression of CYP1A2 from 1 *μ*mol/L onwards and as high as 24-fold changes at 10 *μ*mol/L, the same cannot be said for the corresponding protein expression (Figs. 3, 4). Omeprazole at 50 *μ*mol/L was subsequently used a positive control for CYP1A2 induction in the following experiments with other compounds. Omeprazole produced no effects on the mRNA and protein expression of CYP2D6 but it significantly induced the expression of CYP3A4 at 0.1 *μ*mol/L (<1.5-fold change) and protein expression at 1 and 5 *μ*mol/L respectively. However, these effects were unlikely to be clinically significant for CYP3A4. On the contrary, omeprazole and its metabolite, 5′-*O*-desmethylomeprazole, are found to be time-dependent inhibitors of CYP3A4 (Shirasaka et al. 2013). On the other hand, furafylline is a potent and selective inhibitor of CYP1A2 in humans (Sesardic et al. 1990; Kelly and Sussman 2000). In this study, furafylline attenuated the mRNA CYP1A2 induction activity of omeprazole. These results clearly indicate the reliability of the multiplex RT-qPCR for detecting both CYP1A2 inducers and inhibitors at the transcriptional level.

Currently, there is no known clinical inducer for CYP2D6 at the transcriptional level (Ogu and Maxa 2000; Lynch and Price 2007; Pelkonen et al. 2008). Therefore, no specific control drug was used for CYP2D6. Only quinidine, which is a typical inhibitor for CYP2D6 that inhibits the enzymatic activity of CYP2D6, is used in CYP inhibition studies (Bramer and Suri 1999; Shin et al. 1999). In the RT-qPCR reactions, none of the controls used in this study affects the mRNA and protein expression of CYP2D6 significantly. However, ketoconazole was found to inhibit the mRNA expression of CYP2D6 significantly at high concentration (25 *μ*mol/L). On the other hand, dexamethasone as a positive inducer of CYP3A4 clearly induced the mRNA expression of CYP3A4 at most concentrations tested and dexamethasone at 5 *μ*mol/L concentration was used a positive control for subsequent experiments involving food and nutraceutical compounds. Results for both mRNA and western blot analysis appeared to be consistent in this case. Ketoconazole is shown to bind directly with the iron atom of the heme component within the CYP3A4 active site and is a potent inhibitor of the CYP3A4 enzyme (Ekroos and Sjogren 2006). On the other hand, ketoconazole is also found to inhibit the activation of PXR (Pregnane-X receptor) which is involved in the regulation of CYP3A4 mRNA expression (Duret et al. 2006; Huang et al. 2007). Interestingly, using the multiplex RT-qPCR, ketoconazole behaved like a CYP3A4 inhibitor at the transcriptional level only at a high concentration (25 *μ*mol/L). The CYP1A2, CYP2D6, and CYP3A4 mRNA expression profile observed with the control drugs appeared to correspond well to previous studies. However, the same cannot be said for the western blot analysis. Omeprazole induces the mRNA expression of CYP1A2 but insignificantly for the protein expression. On the other hand, dexamethasone induces both the mRNA and protein expression of CYP3A4 significantly. Western blot appears to be an unreliable method to evaluate CYP protein expression. Recent investigations have revealed a rather poor correlation between mRNA and protein profiles (Ideker et al. 2001; Tian et al. 2004). Translation efficiency, protein half-life, experimental noises, and techniques used prominently influence the correlation between mRNA and protein abundance (Maier et al. 2009).

Andrographolide, a diterpenoid, did not show significant induction effects on all three isoforms using the multiplex RT-qPCR. These results were rather consistent with published results on CYP1A2 and CYP3A4 isoforms. Instead, both ethanolic extract and andrographolide are found to decrease the mRNA expression of CYP1A2, 2C9, and 3A4 in human hepatocytes (Pekthong et al. 2009). As for evaluating the effects of andrographolide on CYP enzymatic activities, Pekthong et al. (2008) derived IC_50_ and *K*_i_ values for CYP1A2, CYP2C9, and CYP3A4 using CYP inhibition assays and utilizing human microsomes. In the CYP1A2 assay, the IC_50_ value for *β*-naphthoflavone (positive control, inhibitor) in human microsomes was 0.085 *μ*mol/L as compared with >100 *μ*mol/L for andrographolide. Similarly for CYP2C9, the IC_50_ value for sulfaphenazole (positive control, inhibitor) was 1.01 *μ*mol/L as compared with >100 *μ*mol/L for andrographolide. As for CYP3A4, the effect of andrographolide was negligible (IC_50_ >200 *μ*mol/L), as compared with ketoconazole (0.11 *μ*mol/L). Andrographolide does not seem to have a significant inhibitory effect on CYP1A2, CYP2C9, and CYP3A4 at the enzymatic level as well.

On the other hand, bergamottin induced the mRNA expression of CYP1A2 from 1 *μ*mol/L onwards with significant copy number values from 5 to 50 *μ*mol/L. The fold changes were ∼11-fold increase at 1 *μ*mol/L and this clearly indicates the CYP1A2 induction properties of this food compound. Our results clearly corresponded with another study, of which a 48 h exposure of cultured human hepatocytes to bergamottin resulted in increased levels of CYP1A2 protein and mRNA (Wen et al. 2002). Bergamottin, a furanocoumarin component of grapefruit juice, is a clinically known mechanism-based inactivator of CYP3A4 and contributes, in part, to the grapefruit juice–drug interaction. Grapefruit juice decreases presystemic metabolism via a mechanism-based inhibition of gut wall CYP3A4 and furanocoumarins are mainly involved in the pharmacokinetic interactions leading to increased side effects and toxicity of coadministered drugs (Diaconu et al. 2011; Lin et al. 2012). However, in this study, bergamottin does not appear to produce significant induction effects on either CYP2D6 or CYP3A4 using the multiplex PCR. Interestingly, bergamottin is a significant in vitro CYP1A2 inducer and in view that the induction could be clinically significant, appropriate clinical trials involving human subjects would be recommended.

Both curcumin and lycopene produce no significant or relevant induction effects on all three isoforms in the HepG2 cells. These results appear to concur with both in vivo (human trial) and in vitro experiments. A standardized curcuminoid/piperine preparation given orally to healthy subjects produced no meaningful changes in plasma C_max_, area under curve, clearance, elimination half-life or metabolite levels of midazolam, flurbiprofen, or paracetamol, indicating that this piperine-enhanced curcuminoid preparation is unlikely to result in a clinically significant interaction involving CYP3A, CYP2C9, or the paracetamol conjugation enzymes (Volak et al. 2013). In another experiment, both curcuma extracts and curcumin did not affect the mRNA expression of CYP3A4 significantly in intestinal cells (Graber-Maier et al. 2010). On the other hand, lycopene was also reported to have no activation potential on PXR-responsive genes like CYP3A4 in HepG2 cells (Ruhl et al. 2004). In addition, lycopene too does not inhibit the cytochrome P450 (CYP) 3A4-mediated drug metabolism (Sunaga et al. 2012). Lycopene, a member of the carotenoid family, found commonly in red pigmented fruit and vegetables is known to have significant antioxidant and pro-oxidant properties and is available in the market as supplements.

Resveratrol, a natural stilbene derivative compound commonly found in red wine or red grape extract is well known for its antioxidant and antiaging activities. It is known to be a mechanism-based inactivator of cytochrome P450 3A4 (CYP3A4) and inhibits significantly human CYP3A4-dependent transformation of cyclosporine, a CYP3A4 substrate (Regev-Shoshani et al. 2004). It is also a well-characterized aryl hydrocarbon receptor antagonist in mammalian cell lines (Aluru and Vijayan 2006). Resveratrol intervention in healthy subjects was found to inhibit the phenotypic indices of CYP3A4, CYP2D6, and CYP2C9 but induces the phenotypic index of 1A2, indicating the potential drug interactions in pharmacological doses (Chow et al. 2010). In the healthy subjects, 4 weeks of 1 g daily resveratrol administration resulted in a 16% decrease in the caffeine/paraxanthine metabolic ratio, suggesting an induction of CYP1A2 activity. It is worth noting that the decrease did not achieve 50% from the baseline, indicating weak to moderate induction. Using this multiplex PCR, only the highest concentration of 50 *μ*mol/L elicited a significant effect on CYP1A2 mRNA expression, although induction was observed as low as 0.1 *μ*mol/L and results appear to correspond with the published data.

In conclusion, a reliable multiplex RT-qPCR can be easily utilized to measure the CYP1A2, 2D6, and CYP3A4 induction properties of food and herbal compounds. Andrographolide, curcumin, and lycopene produced no significant cytochrome induction effects. On the other hand, bergamottin appeared to be a significant in vitro CYP1A2 inducer and resveratrol is a weak one. Examining the cytochrome induction properties of food and herbal compounds help complement CYP inhibition studies and provide proper labeling and safety caution for such products.
